# Mechanical Stimulation

**Published:** 1907-07

**Authors:** W. A. Mathews

**Affiliations:** Hawkinsville, Ga.


### MECHANICAL STIMULATION.

By Dr. W. A. Mathews, Hawkinsville, Ga.

---

In offering a paper on mechanical vibratory stimulation, I have not lost sight of the great good accomplished by chemical therapeutics, nor would I, if I could, lessen the confidence of anyone in drug-therapy.

It is the duty of every physician, to use every reputable method at his command to relieve the suffering of his patients.

I am glad to see the prejudice against other than drug-therapy banishing from the minds of the profession, and carrying with it the once boasted title of "Pill Peddler". Such eminent physicians as Neiswanger, Burdick, Massey, Mornell, Snow and others have proven, and are demonstrating the fact that, the different forms of mechanical therapeutics are entitled to the respect of the profession at large and deserve a place in the armamentarium of every physician and surgeon.

They have wrested these methods from the hands of the quack and demanded for them their proper recognition from the "ethical."

So today they are going hand in hand with surgery and other therapeutic methods.

In order to study vibration as a therapeutic agent, it is necessary to briefly consider the spinal cord, with its relation to the sympathetic nervous system.

I am aware of the fact that, a class of irregular practitioners called "Osteopathist" treat every manner of disease by manipulating the spine, which causes the profession to look with suspicion upon anything that savors of their method.

Such objections however, are unreasonable from the fact that, they did not discover the spinal cord nor its functions, or its relation to, or influence over diseased organs. Because they attempt to manipulate the nerve centers in the cord, does not constitute a valid reason why physicians should refuse to make it their field of operation if conducted upon scientific principles.

If there be any merit in the so-called osteopathy, it should be appropriated by the medical profession and applied scientifically to the treatment of diseases in which it is indicated, and not lauded as a cure-all for all manner of ills, by a class of irregulars who never studied the science of medicine nor the pathology of diseases, except from this one view-point. The spinal cord is an elongation or continuation of the brain, and consists of an interior, posterior and two lateral colums. From the grooves from between the interior and lateral columns arise the anterior or motor roots of the spinal nerves.

The roots of the posterior or sensory nerves take their origin from between the lateral and posteria columns, so on each side of the vertebrae forming the spinal column is given off a root or spinal nerve: about thirty-two pairs.

There is on each side along the front of the vertebral column, a chain or ganglion of nerve branches formed of filliments from the spinal nerves with which they are in relationship throughout the cord.

So there is a pair of sympathetic nerves correspondiug with each pair of spinal nerves. This series of ganglia compose the centre of the sympathetic system, which is connected with the vicera by nerves passing from this chain, or central part, to plexuse formed about the vessels or within the substance of the viscera, which contain numerous small ganglia. These small ganglia are not mere aggregations of nerve cells, but are composed of grey matter and are claimed by many physiologists to be the centres of impressions and receivers and transmitters of intuitive knowledge.

Keeping in mind these few anatomical facts about the spinal cord and its relation to the sympathetic nervous system, its functions may be summerized as follows:

1st., It is the principal seat of reflex nerve action.

2nd., It is the centre of the vaso-motor system.

3rd., It influences arterial tone and the action of the various vicera.

4th. It is an index to abnormal conditions iu many parts of the body.

This fourth proposition is generally accepted, by those not using physicial methods with a grain of salt; nevertheless, it is a constantly demonstrated fact to those using the methods that when there is a disturbance in any organ or area, it matters not how far removed, there will be a corresponding irritation at the spine of the nerves controlling nutrition or muscular action of the parts affected.

In chronic diseases of any organ or viscera the muscles over the reflexly affected spinal nerve centres will be contracted or atrophied in proportion to the length of time the condition has existed.

This is explained by the fact that, the muscles referred to get their nerve supply from the same area in the cord that is reflexly affected from the diseased viscera, and therefore, participate in the irritation to which they give rise. When the disease is of very great intensity or of long standing, the nerve centres are proportionately inhibited and lose their power to normally functionate without added stimuli, which should be artificially applied directly over the affected centres.

For illustration and evidence of the above facts, it is noted that, irritation of the nerves along the third and fourth cervical will cause a disturbance of the visual organs; so will abnormal conditions in the eye cause an irritation of these spinal nerves. Again, a diseased liver will be associated with sensitiveness at the roots of, from the third to the tenth dorsal nerves.

If these statements seem to any one like the products of an overdrawn imagination, let him submit them to the crucible of practical test, and it will soon be his rountine pratice to carefully examine the spine for points of contractures, or sensitiveness as an index to a diseased organ or vicera and when found if properly vibrated the full benefit of mechanical therapy will be manifested to a degree not otherwise possible.

Vibratory stimulation is found from experience to be capable of:

1st., Increasing nutrition, improving muscular and general matabolism and in the production of animal heat.

2nd., Stimulating the secretory and excretory organs, thereby improving nutrition and assisting in elimination.

3rd., Softening and relieving muscular contractures, lessening engorgement and congestion.

4th., Facilitating the removal through the lymphatics of exudates and other products of inflammation.

5th., Of inhibiting and relieving pain.

The above may seem to indicate that, those using vibration as a therapeutic agent, consider it a cure-all or an exclusive antidote for every abnormal physicial condition; but not so, yet we know of no other agent, either physical or chemical, that will give effective service in obtaining the above results with as few disappointments as vibration properly applied.

The treatment is rational and scientific and is based upon sound physiological principles. The theory being that, all the functions and organs of the body are controlled by certain nerve centres, located principally along the spinal cord and when a disease exists, if these centres are reached and treated by either stimulation or sedation, (either can be done by vibration), the diseased organ or viscera will in most cases be restored to normal action.

It is an accepted fact that, all physical life, from the smallest cell to the universe as a whole, is in a constant state of vibration, and in the body the nerve fibres and cords are more highly sensitive than the other tissues, yielding to outside influences and conveying impressions to the different parts, either stimulating or depressing their normal action.

The application of vibratory stimulation does not antagonize nature, nor interfere with the elaboration of physiological processes, but on the contrary supplements her efforts and aids her in overcoming these outside influences. It can be administered with less deleterious effects than any other agent, stimulation here and there a flagging organ or inactive function, putting such organism in a condition, as to render "restitutio ad intregrum" possible, where otherwise it would be impossible.

We are not forgetful of the fact that, the real power of repair resides only with nature, yet when she falters or is hindered in performing her function, we should come to her relief with methods as near like hers as possible, so if every function and the action of every organ is dependent upon their nerve supply and the nerve force depends upon its state of vibration and is susceptible to artificial stimulation, then this method is the most natural one that can be applied.

In applying vibration patients should remove heavy and tight clothing and be placed in a prone position upon a firm padded table, or couch without springs, and be instructed to completely relax every muscle during treatment.

The length of stroke, the degree of pressure and duration of time that treatment should be applied to a given point, will depend upon the condition and location of the area affected, the idiosyncrasies of the patient, as well as the results desired.

As a general rule mild stimulation will require from four to eight seconds, vibratory stimulation from ten to fifteen seconds and inhibitory effects from fifteen to twenty-five seconds.

Mild stimulation may be applied along the spine with brush attachment, when it is only desired to increase the blood supply to a given area for tonic effect, but when there is severe pain or pronounced atony or inactivity, treatment should be applied between the transverse processes with ball attachment, using heavy stroke and deep pressure.

The instrument used should be of the rigid arm variety.

It is not possible to obtain deep penetration of the impulses with a small light instrument with a flexible shaft.

In my opinion it amounts to nothing more than massage of the integument, which is beneficial only in a few local conditions, such as slight injuries, œdemas, and impaired peripheral circulation affecting the superficial structures. It does not penetrate to the controlling nerve centres and therefore, cannot influence the general system to any extent.

I shall mention a few conditions in which vibration gave excellent results, as indicated by a tabulated report of cases treated during a period of six months in the clinical department of the Vibrator Instrument Co., of New York City. Out of nine cases of functional impotency there were nine cures (100 per cent), treatment requiring from two to seven weeks.

Out of over two hundred and fifty cases of constipation there were about ninety-five per cent. of cures. In Amenorrhea, there were about seventy-five per cent. cured, while the remainder were much benefited, (this does not include cases where there were mechanical obstructions) in dismenorrhea, associated with sensitive spines, all were practically cured, while those not accompanied by sensitive points along their spines were only benefited after a long course of treatment.

All myalgic conditions yielded to only a few treatments, while in neuritis and paresthesia it was shown to be almost a specific.

With my own limited experience of two years, using a Chattanooga Vibrator, I have cured many cases in which drugs had failed, while others I have checked the progress of the disease and gave the patient longer lease on life.

My results compare closely to those of the clinic referred to.

My best successes have been in treating constipation, impotency, neuralgias, except of the head and face, muscular rheumatism, insomnia, amenorrhea, dismenorrhea, melancholia and hysteria, while general debilitated conditions where no definite cause can be located, are invariably benefited. I have completely removed several goiters of the simple variety and reduced about one-half those of the exophthalmic variety.

In locomotor-ataxia a good per cent. show signs of marked improvement, regaining control of the sphincters with improvement in gait, diminution of pain and the return of sensation to the limb.

As a pain relieving agent where there is doubt as to its cause the vibrator is a most effective agent.

In many obscure cases it is a valuable aid in making and confirming diagnosis.

